# Two psammophilic noctuids newly associated with beach plum, *Prunus maritima* (Rosaceae): The Dune Noctuid (*Sympistis riparia*) and Coastal Heathland Cutworm (*Abagrotis benjamini*) in Northeastern North America (Lepidoptera, Noctuidae)

**DOI:** 10.3897/zookeys.661.10867

**Published:** 2017-03-14

**Authors:** Paul Z. Goldstein, Michael W. Nelson

**Affiliations:** 1 Research Entomologist, Systematic Entomology Laboratory, USDA, National Museum of Natural History, E-502, P.O. Box 37012, MRC 168, Washington, DC 20013-7012; 2 Invertebrate Zoologist, Natural Heritage & Endangered Species Program, Massachusetts Division of Fisheries & Wildlife, 1 Rabbit Hill Road, Westborough, MA 01581

**Keywords:** *Abagrotis*, *Sympistis*, beach plum, *Prunus maritima*, Noctuidae, psammophile

## Abstract

Beach plum, *Prunus maritima* Marshall, 1785 not Wangenh., 1787 (Rosaceae), currently under development as a potential crop, represents an under-acknowledged host plant for several Lepidoptera that have undergone declines in the northeastern USA. The Coastal Heathland Cutworm, *Abagrotis nefascia* (Smith, 1908), and the Dune Noctuid, *Sympistis riparia* (Morrison, 1875), are unrelated species of psammophilic noctuines (Lepidoptera: Noctuidae) regularly encountered on a localized basis in coastal southern New England and New York, and whose precise life history requirements are undocumented. We inferred and, based on field observation and rearing, corroborated beach plum as a larval host for these species in Massachusetts; the plant’s role in sustaining other moths with limited or contracting regional distributions is discussed. *Sympistis riparia*, belonging to a widely distributed complex of closely related species, has been associated specifically with both maritime and freshwater dunes. The eastern populations of *Abagrotis nefascia* represent a conspicuous range disjunction, separated from the nearest western populations by more than 2000 miles, and originally described by Franclemont as race benjamini of *Abagrotis crumbi*, both later synonymized with *Abagrotis nefascia*. Following examination of types and other material, an evaluation of putatively diagnostic features from both the original description and our own observations, genitalic characters, and the results of provisional barcode analyses, *Abagrotis benjamini* Franclemont, **stat. rev.**, is elevated to the rank of a valid species rather than representing eastern populations of *Abagrotis nefascia* (=*crumbi*) to which it originally referred.

## Introduction

Distributions of herbivorous insects are often constrained by ecological or edaphic factors, in addition to the presence of their host plants. In northeastern North America, some of the most threatened terrestrial plant communities, including pitch pine-scrub oak barrens, maritime heathlands, shrublands, and grasslands, are limited to sandy, well-drained soils geologically derived from glacial outwash or ancient lake beds, sometimes collectively referred to as sandplains. Lepidoptera associated with such soils are often localized and hence prominent among those considered regionally endemic or locally threatened, and in the Northeast are heavily concentrated in coastal areas. Habitat associations and life history requirements of many sandplain insects remain undocumented, potentially due to a combination of regional variation in host specificity and the fact that observations of adult moths may belie the precise location of their larval hosts. Dunes and related habitats, such as barrier beaches, are obvious features of coastal areas, often adjacent to strictly terrestrial sandplain habitats, but are commonly overlooked as potential resources for invertebrates and may further confound our inferences of host associations. In several cases, moths’ habitat associations, if not host plants themselves, have been inferred or asserted in the absence of corroborating life history information, even to the point of being reflected in the animals’ common names.

The Dune Noctuid, *Sympistis riparia* (Morrison), and the Coastal Heathland Cutworm, *Abagrotis nefascia* (Smith), are cases in point. Both represent large noctuid genera with numerous cryptic species (Lafontaine 1998; Troubridge 2008) variously associated with Ericaceae, Rosaceae, and other “low plants” (cf. Crumb 1956). The highly speciose genus *Sympistis* is composed of several distinct groups of variously cryptic and localized species. The smaller genus *Abagrotis* includes a range of benign and pest species, many of them widespread, particularly in western North America. Both *Sympistis riparia* and *Abagrotis nefascia* are considered widespread in North America but in the Northeast are heavily concentrated in or confined to coastal areas, where they are observed in association with a narrow range of plant communities primarily on sandy outwash soils. Finally, the habitat associations of these two species were thought to be well-enough understood to have yielded common names referencing dunes and maritime heathlands, respectively.

The range of *Sympistis riparia* extends westward to California and British Columbia, and although the eastern portion includes sporadic inland occurrences and coastal areas from New Brunswick to the mid-Atlantic, records are most heavily concentrated along the southern New England coast and the southern shores of the Great Lakes. The distribution of *Abagrotis nefascia*, in contrast, is more conspicuously centered in western North America, where it is considered a major pest of grapes (Lowery and Mostafa 2010), and the species’ disjunct occurrence in the Northeast is confined almost entirely to coastal areas from New Brunswick to southern New England and New York, with the nearest conspecific populations roughly 2000 miles away. The northeastern populations were described under the trinomen Abagrotis crumbi race benjamini Franclemont 1955, and later synonymized with *nefascia* by Lafontaine (1998: 221) along with nominate *crumbi*. Because of their ecologically and geographically narrow distributions in the Northeast, both *Sympistis riparia* and *Abagrotis nefascia* are listed as species of conservation concern in one or more New England states, including Massachusetts, where they represent two of three state-listed noctuids whose host plant associations have not been documented (the other being *Apamea inebriata* Ferguson, a possible associate of *Andropogon glomeratus* (Walt.) Britton, Sterns & Poggenb.).

This project began with the modest goal of elucidating the life history and host plant of *Sympistis riparia*, which we all-too-slowly grew to suspect included one of the most common dune shrubs, beach plum (*Prunus maritima*). Well-known regionally for its fruit, this shrub has a checkered history of cultivation (Uva 2003), but has recently returned to the fore under development as a potential crop (Uva and Whitlow 2007). Although beach plum is recorded as a host for several oligophagous insects (bees as well as moths), herbivores of *Prunus* are in general not known for strictly monophagous feeding habits. Having guessed that any putative botanical specialization on the part of *Sympistis riparia* was as much a function of substrate as plant chemistry, our efforts were focused accordingly on areas in the vicinity of dune systems already familiar to us for their unique occurrences of insects. These efforts led in turn to our examination of *Abagrotis* in association with beach plum. Our purpose here is thus to document our observations of host use for both species and clarify the taxonomic status of eastern *Abagrotis nefascia* based on type material as well as reared specimens and DNA barcode data.

## Materials and methods

### Larval and adult surveys

Despite its well-known association with both maritime and inland dunes, the host(s) of *Sympistis riparia* have eluded lepidopterists for decades. Among the candidate host plants suggested on the basis of their prevalence in dunes was American beachgrass (*Ammophila breviligulata* Fern.), which would account for a high concentration of records along the Atlantic Coast as well as the shores of the Great Lakes. Beachgrass represents not only an important structural and stabilizing component of dunes but an important host for herbivorous insect biodiversity, including other noctuid dune associates such as *Apamea lintneri* (Grote, 1873) (Quinter 2009: 103). With over 190 species in North America, *Sympistis* represents the most speciose noctuid genus on the continent, but relatively few species have documented larval life histories or host associations. Those which are known involve broad-leaved plants including Scrophulariaceae, Ericaceae, Caprifoliaceae and most particularly Rosaceae. Given this background, an association with a grass seemed highly unlikely: graminivory in Lepidoptera tends to be either highly conserved or associated with polyphagous feeding habits not known from any species of *Sympistis* Feeding on grasses requires a complex of behaviors and morphological features necessary to consume and digest siliceous plant material, a suite of attributes unlikely to evolve frivolously. To our knowledge, no isolated species of graminivores are known to be nested within groups of folivores of broad-leaved plants, and an isolated *Ammophila*-feeder within a lineage dominated by Rosaceae feeders would be unique for Noctuidae, if not Lepidoptera.

With this in mind, we began our survey of potential host plants with what we surmised to be more likely candidates, the shadbushes or serviceberries *Amelanchier* Medikus. *Amelanchier* was also among the hosts recorded by Crumb (1956: 121) for *Abagrotis nefascia* (as *Abagrotis* n. sp.) (Lafontaine 1998). Although we recognized that *Sympistis riparia* is unlikely to be strictly monophagous, it seemed plausible that the local distribution of *Amelanchier* in a range of coastal habitats, and the wider occurrence of various *Amelanchier* species would account for both the regional distribution of *Sympistis riparia* and the more diffuse local distribution of adult moths. Based on our understanding of other *Sympistis*, we expected *riparia* to overwinter in the egg stage, and given that adults had been recorded as early as mid-June, we expected that a narrow larval development period was accommodated by feeding, at least initially, on floral tissues available in early spring. In this region, *Amelanchier* is the first flowering rosaceous shrub of the season, referred to as “shadbush” for the coincidence of its flowering with the return of shad to their freshwater spawning grounds.

**Figure 1.**
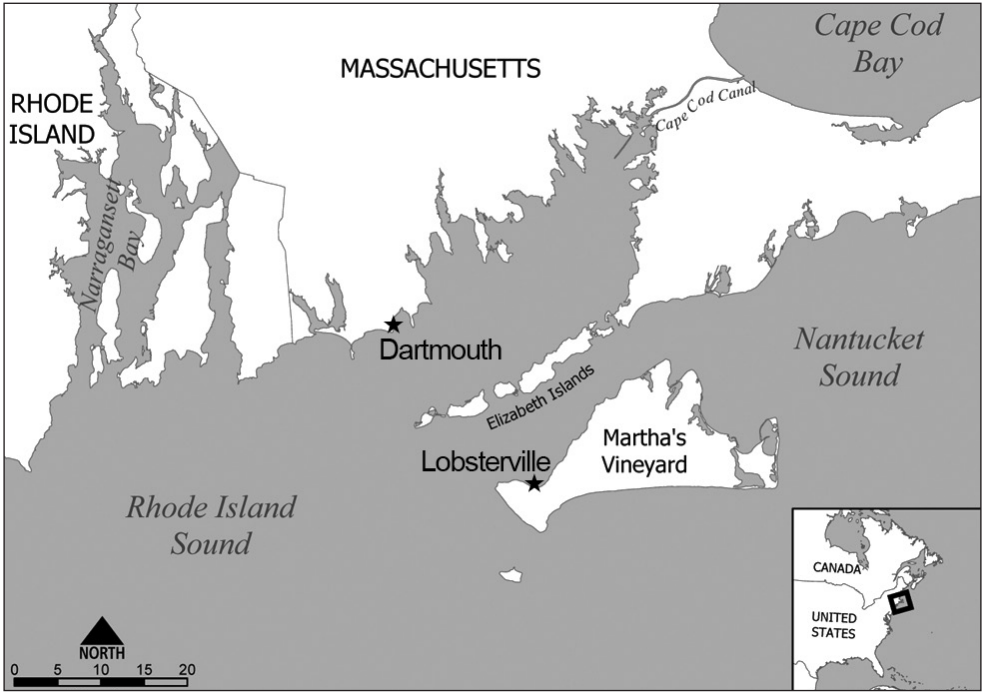
Section of coastal southern New England including Cape Cod and the Massachusetts offshore islands. Study sites in Aquinnah and Dartmouth marked with a ★.

**Figure 2.**
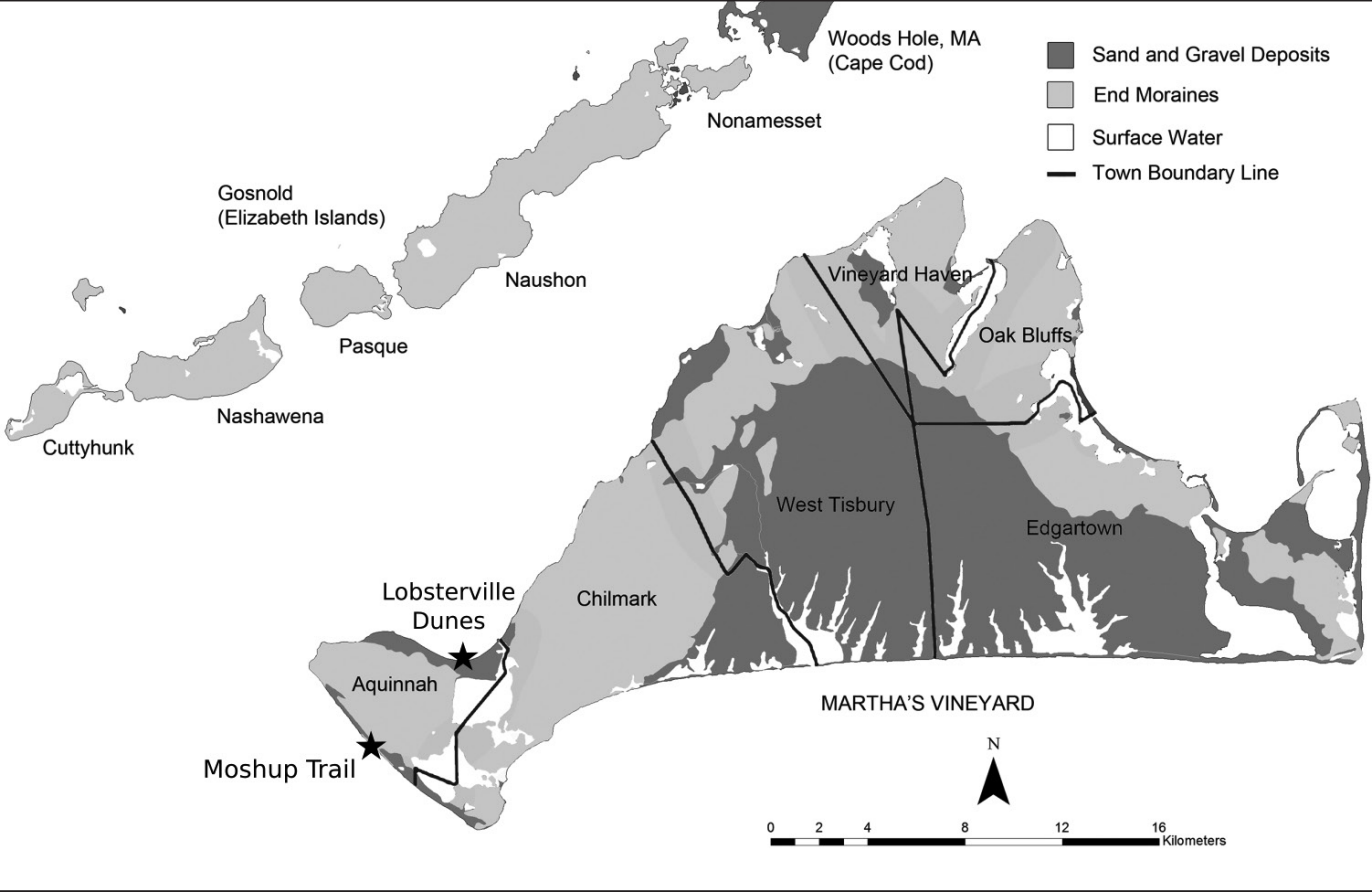
Martha’s Vineyard and neighboring islands comprising Dukes County with indication of town boundary lines and relevant geologic formations and substrates overlain. Moshup Trail and Lobsterville Dunes sites marked with a ★.

**Figure 3.**
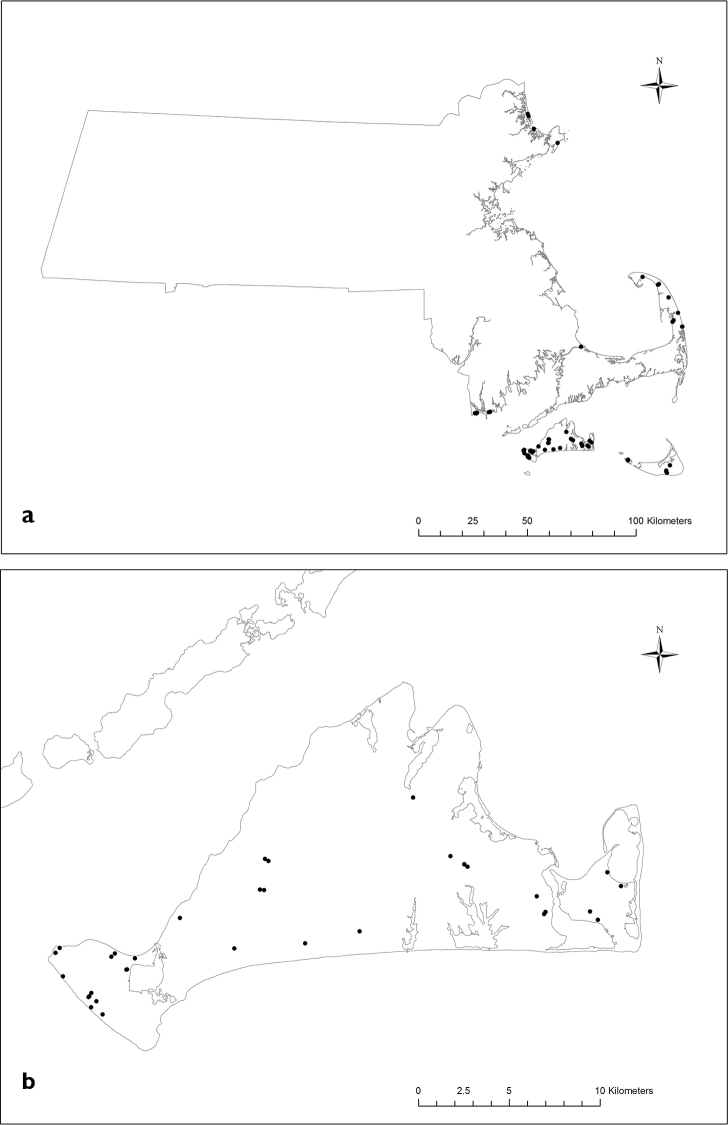
**a** Distribution of *Sympistis riparia* adults recorded from Massachusetts as of 2016 (MNHESP), **b** Distribution of *Sympistis riparia* adults recorded from Martha's Vineyard as of 2016 (MNHESP).

**Figure 4.**
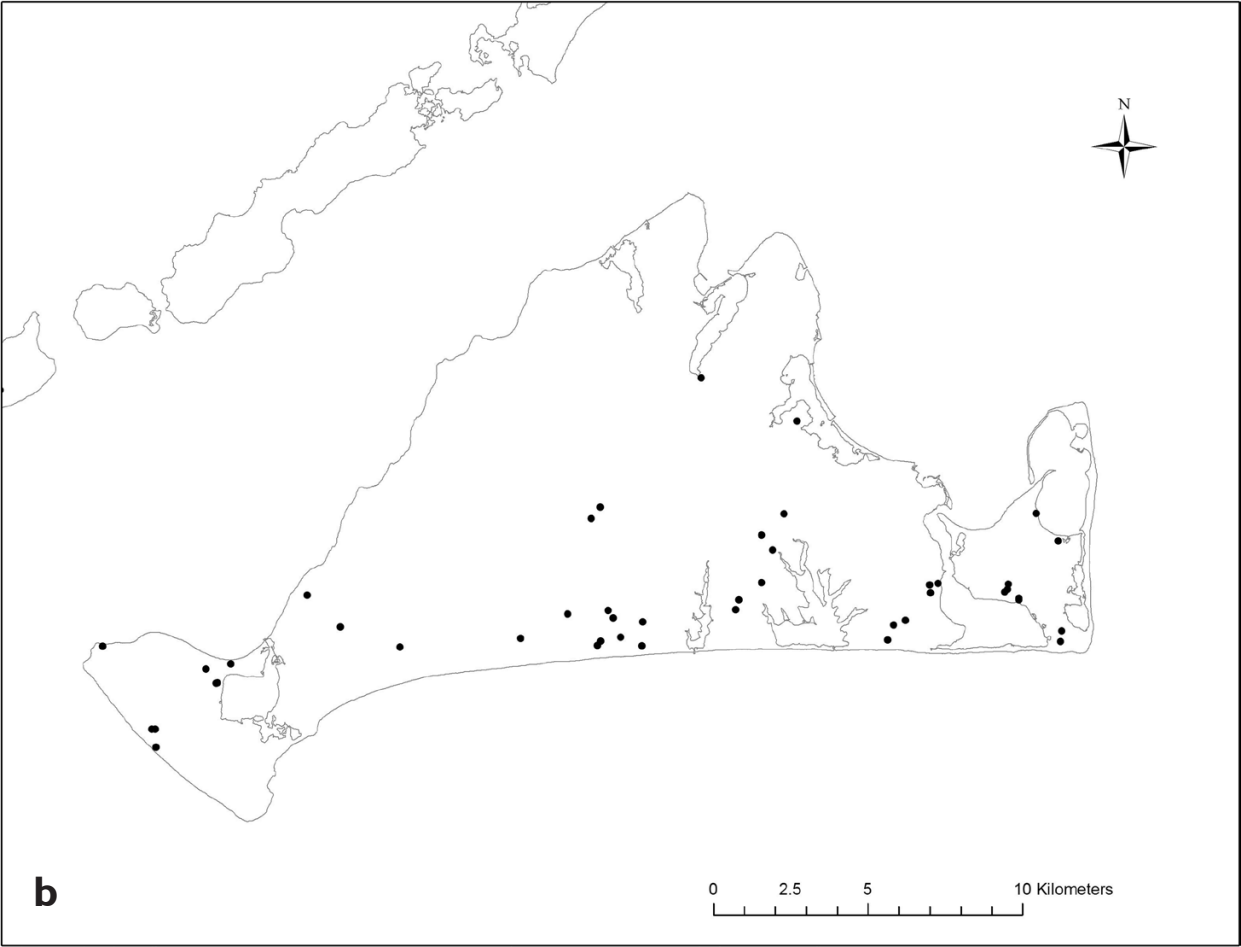
**a** Distribution of *Abagrotis benjamini* adults recorded from Massachusetts as of 2016 (MNHESP), **b** Distribution of *Abagrotis benjamini* adults recorded from Martha’s Vineyard as of 2016 (MNHESP).

As a state-listed species, observations of *Sympistis riparia* are tracked by the Massachusetts Natural Heritage Program, and upon examining these data we initiated our survey at a series of sites near Moshup Trail in Aquinnah, Massachusetts (Figs 1–2), which at the time supported the highest known concentration of recent observations (Fig. 3a, b). This geologically unique area comprises a structurally complex mosaic of morainal heathlands, shrublands, and perched fens enclosing migrating dunes interspersed with cranberry (*Vaccinium macrocarpon* Aiton), sundew (*Drosera intermedia* Hayne) and bushy bluestem (*Andropogon glomeratus*). This unusual admixture of plant communities supports a unique assemblage of Lepidoptera (Goldstein, in prep.), including a number of regionally localized or unique occurrences. Among those species that had been recorded in significant numbers was *Abagrotis nefascia*, but given its wider distribution both in Massachusetts and on Martha’s Vineyard (Fig. 4a, b), we did not initially suspect it shared a primary host plant with *Sympistis riparia*.

**Figures 5.**
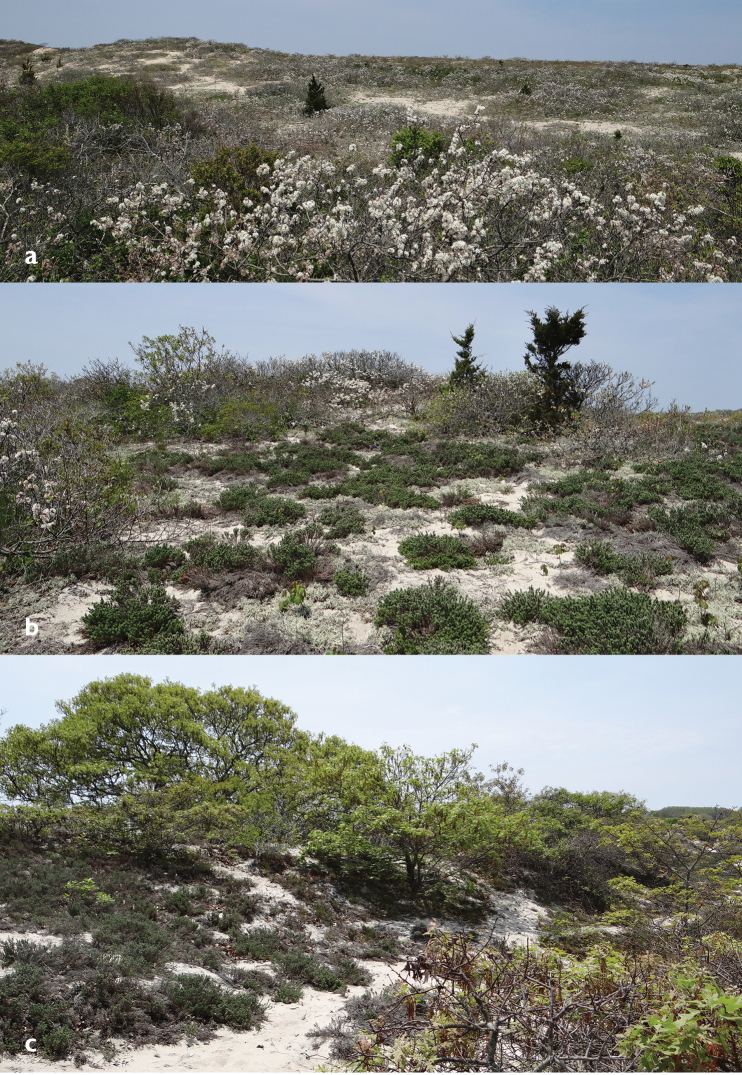
Examples of habitats represented at the Lobsterville Dunes. **a** Immediate backdune, dominated by beach plum, also in foreground **b** Floristic diversity on a secondary dune, the vegetation including beach heather (*Hudsonia tomentosa*) and heavy lichenization **c** A section of dune supporting a mixture of salt- and wind-dwarfed oak, including scrub oak (*Quercus ilicifolia*) in the foreground.

**Figures 6.**
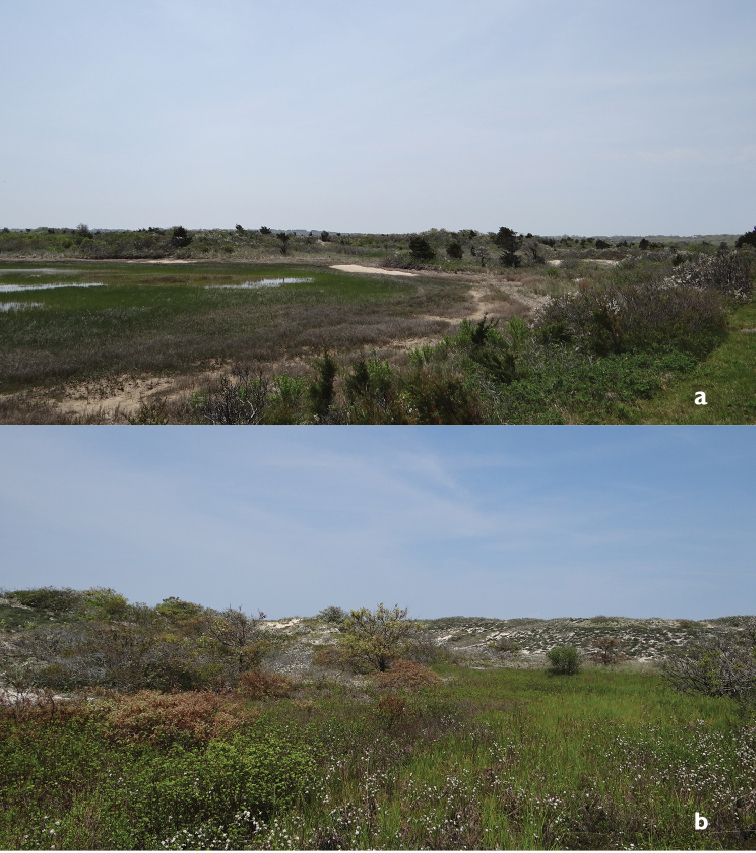
Examples of wetlands interspersed among the Lobsterville Dunes. **a** Graminoid wetland edged in part with Atlantic white cedar (*Chamaecyparis thyoides* (L.) Britton, Sterns & Poggenb.) **b** Backdune wetland supporting a colony of black chokeberry (*Aronia melanocarpa* (Michx.) Elliott).

A casual survey for *Sympistis* larvae on *Amelanchier* in April 2011 was timed to coincide with the plant’s flowering season, but was unsuccessful. Later the same season, however, light trapping efforts at a site approximately three kilometers distant yielded adult moths in high numbers which were unprecedented in our experience. Although radically different from our initial target site in terms of its immediate plant community composition, which includes a mixture of mesic forest and morainal scrub, the trap site overlooks the expansive and under-studied peninsular dune system, Lobsterville Dunes. This site supports a structurally and floristically diverse vegetative cover lichenized and dominated by beach plum *Prunus maritima*, scrub oak *Quercus ilicifolia* Wangenh., and beach heather, *Hudsonia tomentosa* Nutt. (Fig. 5a–c), and pocketed with numerous graminoid and shrub-dominated wetlands and interdunal swales (Fig. 6a, b). The observation of high numbers of *Sympistis riparia* in view of large, highly visible stands of beach plum led to the sudden, strong, and overdue suspicion that one of the most obvious native rosaceous dune plants had been overlooked as a potential host. It also led to our initiating survey efforts targeting beach plum caterpillars beginning the following spring (2012), at which time regular light trapping was also begun within the Lobsterville dune system itself, in parallel with ongoing light trapping at Moshup Trail. This was intended to evaluate whether *Sympistis riparia* did indeed occur in consistently higher numbers within the strict confines of the dune system than had been observed previously. Over 100 light traps were deployed in Aquinnah, including 25 at Lobsterville Dunes between 2012 and 2016. Many specimens were prepared for study, and others frozen in liquid nitrogen for future molecular work.

### Morphological examination of *Abagrotis*

Pinned specimens were examined with an incandescent light source. Genitalic preparations varied, the more recent ones following parts of Clarke (1941) and Lafontaine (2004), but using chlorazol black and mounted in euparol, the vesicae everted in water and fixed in ethanol. The most recent dissections were made following an overnight room-temperature soak in supersaturated sodium hydroxide, and examined prior to mounting. Slide preparations were examined with dissecting and compound microscopes. Photographs were made using the Microptics and Visionary Digital imaging systems and images manipulated with Adobe Photoshop® (Adobe Systems, Mountain View, CA). Measurements were made with the aid of an ocular micrometer. Forewing length was measured from the center of the axillary area up to the apex of the forewing (FW). Terminology follows Lafontaine (1998, 2004).

## Systematics

### Status of *Abagrotis nefascia* in the Northeast

Even a cursory examination of the known distribution of *Abagrotis nefascia* reveals an obvious disjunction between the range occupied by *nefascia* west of the Rocky Mountains and the populations restricted to a narrow band along the northeastern coast of North America. Recognizing this, Franclemont (1955) attributed the north eastern populations as an infraspecific race (*benjamini*) of *Abagrotis crumbi*, which he described based on material reared by S.E. Crumb. Buckett retained both names and their ranks in his (1969c) revision of *Abagrotis* [part], but designated a specimen of *Abagrotis crumbi* as the lectotype of *Abagrotis nefascia* (Figs 13–18). Lafontaine synonymized both *Abagrotis crumbi* and *Abagrotis crumbi benjamini* with *Abagrotis nefascia*, having traced considerable confusion surrounding the identity of *Abagrotis nefascia* involving no fewer than four specific epithets: *Abagrotis nefascia* (Smith, 1908), *Abagrotis forbesi* (Benjamin, 1921), *Abagrotis crumbi* Franclemont, 1955, and *Abagrotis reedi* Buckett, 1969. As Lafontaine (1998: 221) recounts, *Abagrotis nefascia* had been predominantly associated with specimens referred to *Abagrotis reedi* by Buckett (1968b), who synonymized *Abagrotis forbesi* with *Abagrotis nefascia*. Although Buckett (1968b: 19) designated a lectotype of *Abagrotis nefascia*, the specimen is conspecific with *Abagrotis crumbi* and not with *Abagrotis forbesi* as he had supposed. Further confusing matters, the lectotype genitalia that was figured by Buckett (1969: fig. 88) included the aedeagus of the *Abagrotis nefascia* lectotype, but the mislabeled valvae belonging to a specimen of *Abagrotis forbesi*. In disentangling these threads, Lafontaine reversed Buckett’s synonymy, revived *Abagrotis forbesi* as a valid species and synonymized both *Abagrotis crumbi* and Abagrotis crumbi race benjamini with *Abagrotis nefascia*. The history of nomenclatural confusion, including the misspelling of *Abagrotis nefascia* as *Abagrotis negascia* in the original description, is summarized by Lafontaine (1998: 221): “*Nefascia* was treated as a senior synonym of *forbesi* by Buckett (1968c [cited herein as 1968b]) but the lectotype designated by Buckett (1968c[b]: 19) is clearly referable to the species previously known as *crumbi*. The valve shape and presence of a secondary diverticulum in the subbasal diverticulum of the lectotype are diagnostic for *nefascia* (=*crumbi*).”

Those populations formerly known as Abagrotis crumbi race benjamini have thus not been validly recognized as taxonomically distinct since 1998. Despite the formal synonymy, *benjamini* has been retained as a subspecies of *Abagrotis nefascia* outside the taxonomic literature, most prominently in the roster of protected species and subspecies in New York (New York Natural Heritage Program 2016), and Connecticut (Connecticut Dept. of Energy & Environmental Protection 2016) as *Abagrotis nefascia benjamini*, as well as at iNaturalist.org. This usage appears traceable to entries in NatureServe (http://explorer.natureserve.org/), a portal for disseminating information on taxa of conservation concern. Below we revisit the grounds for retaining or elevating the status of *Abagrotis benjamini* based on available character information, and revise its status accordingly, providing a diagnosis and re-description of the genitalia. A more detailed treatment of the larvae and life history of these animals will occupy a separate work.

Franclemont wrote in his description of race benjamini (1955: 46): “Similar to the typical race, but the markings are a little less distinct, and the color is generally duller. All the specimens are a rather uniform tannish brown with a contrasting purplish gray terminal area.” He described the male genitalia as “somewhat larger than the typical race” and the female as having the “ductus bursae more heavily sclerotized; general characteristics similar to those of *forbesi*.” The paratypes of *benjamini* comprise specimens from East New York [Brooklyn], NY; Connecticut; and Martha’s Vineyard, MA. Upon close examination of these and other specimens, including a series of Massachusetts specimens and the holotypes of *Abagrotis crumbi*, *Abagrotis nefascia*, *Abagrotis forbesi*, and *Abagrotis reedi*, we noticed consistent, if subtle, differences between eastern specimens and typical *Abagrotis nefascia*. In addition to those differences in coloration noted in Franclemont’s (1955) description, we focus on the configuration of spines on the vesica as well as characters adduced by Franclemont (wing pattern and sclerotization of the female ductus), Buckett (number of signa on the bursa copulatrix), and Lafontaine (secondary diverticulum in the male vesica).

Buckett (1968c) characterized *Abagrotis crumbi* in a different species group than *Abagrotis nefascia* [sensu (Buckett 1968b), not *Abagrotis nefascia* (Smith, 1908)]. Although he accurately described the terminal area of the *benjamini* forewing as fading inwardly, he characterized both *Abagrotis nefascia* and *Abagrotis crumbi* as lacking heavily sclerotized ductus bursae and, more importantly, described the bursae of *Abagrotis crumbi* and *benjamini* as having two signa, as in *Abagrotis alternata* (Grote, 1864) (Buckett 1968c: 4) versus a single signum on the bursa of *Abagrotis nefascia* (sensu Buckett 1968b: 19). We failed to observe more than one signum on any Massachusetts specimen; most of those we examined from the type series of *Abagrotis crumbi*. All the more recently collected western *Abagrotis nefascia* possessed two.

As Lafontaine suggested, subtle features of wing pattern may be unreliable indicators of phylogenetic affinity in *Abagrotis*. That said, both Franclemont and Lafontaine acknowledged trends in coloration and shading among eastern *Abagrotis nefascia* that may serve to differentiate most specimens from their western counterparts. Eastern specimens range in forewing ground color from a gray brown to brick red; we have not seen any with a dark-maroon forewing ground color.

In addition to exhibiting the secondary diverticulum putatively diagnostic for *Abagrotis nefascia* (Lafontaine, 1998: 221), the scobinate plate on the vesicae of eastern specimens tends to be less evenly denticled. The basal sclerotized patch consists of a more heteroideous series of teeth, with two or three very prominent and 5–8 much smaller (Figs 19–23). Specimens with the most conspicuous teeth tend to have them arranged in a nearly co-planar fashion, almost giving them the appearance of a circular saw blade. Western specimens typically have more than 12 teeth comprising a more graduate series of size, and always arranged in multiple rows.

Finally, we observe that the presence of signa on the female bursa has been interpreted inconsistently in this group. Buckett (1969b: 19) specifically recorded *Abagrotis nefascia* as “possessing a single signa [sic]” and *Abagrotis crumbi* (as a member of the *Abagrotis alternata* species group; Buckett 1969c: 4) as “possessing two signae [sic].” Lafontaine (1998: 205) described *Abagrotis* females as having the “bursa lacking signa...or with two signa.” In the material we examined, we noted the presence of two signa, although one highly reduced, in both *Abagrotis nefascia* and *Abagrotis crumbi*, *contra* Buckett (1969c) (Fig. 24). However, we failed to observe a second signum, reduced or otherwise, on the bursae of ten female *Abagrotis benjamini* examined.

### Barcode data

Interspecific variation in COI sequences in *Abagrotis* is atypically low for a noctuid genus. In the analyses of Zahiri et al. (2014), 29 of 248 genera examined account for 101 identified examples of interspecifically shared haplotypes, with *Abagrotis* accounting for more such incidents (eight) than any other genus examined except *Euxoa* (17). Therefore intraspecific divergences of less than 1% are typically considered more significant within species of *Abagrotis*. Our Massachusetts specimens, however, shared sequences with specimens from New Brunswick, and these collectively differed from a cluster of reliably determined specimens comprising several species with shared haplotypes, including *Abagrotis nefascia*.

We typically eschew the use of distance-based analyses of character data as rigorous arbiters of either phylogenetic relationship or species diagnosis (Goldstein and DeSalle 2011). Such interpretations emulsify potential diagnostic data by masking the visualization of character state distributions on trees, and contradict principles through which statements of monophyly can be legitimately tested, especially for data as minimal as the short sequence conventionally used in DNA barcoding efforts. Instead, we view distance analyses of DNA barcodes as provisional estimates of potential species boundaries, and employ a more transparent cladistic analysis for the limited purpose of summarizing and visualizing character state distributions. In this case, the clustering of eastern “*nefascia*” from Massachusetts and New Brunswick is corroborated by a unique combination of thirteen character state changes, greater than the combined interspecific variation among specimens of seven species in the adjacent cluster. We note an analogous cluster of specimens from British Columbia determined as *Abagrotis nefascia*.

### Observations of adults and larvae

In Aquinnah, high numbers of adult *Sympistis riparia* were observed, routinely exceeding 200 specimens in a single light trap, the highest numbers appearing between 21 June and 5 July. Based on our capture dates, the flight season of *Sympistis riparia* is somewhat protracted, extending from mid-June through early August. Females retained for obtaining eggs oviposited with varying success, most readily when presented with honey water and in the presence of beach plum foliage. Eggs proved difficult to overwinter without succumbing to desiccation or mold but efforts involving chilled hydration and aureomycin-laden artificial diet are ongoing.

Other species appearing in unusually high numbers included *Abagrotis benjamini*, the erebids *Argyrostrotis anilis* (Drury, 1773), *Catocala herodias* Strecker, 1876, and *Drasteria graphica* Hübner, 1818; and the noctuids *Schinia spinosae* (Guenée, 1852) and *Eucoptocnemis fimbriaris* (Guenée, 1852). Species apparently associated with beach plum included regular occurrences of Wild Cherry Sphinx (*Sphinx drupiferarum* J.E. Smith, 1797), which has declined in the Northeast (Wagner, 2012), and now appears confined to coastal areas (Goldstein et al. 2015). During the course of our work we observed other widespread but often scarce *Prunus*-feeding moths that have declined. In particular, both adult *Hyalophora cecropia* (Linnaeus, 1758) and cocoons on beach plum were observed frequently. Of equal ecological interest were the high numbers of moths associated with host plants other than *Prunus* or other signature dune species, and in particular species associated with scrub oak such as *Catocala herodias* and *Cicinnus melsheimeri* (Harris, 1841) (Mimallonidae).

Finally, we collected at least four species of *Abagrotis* in addition to *Abagrotis benjamini*, namely *Abagrotis alternata*, *Abagrotis brunneipennis* (Grote, 1875), *Abagrotis cupida* (Grote, 1865), and *Abagrotis magnicupida* Lafontaine, 1998, some of which have since been reared as wild-caught larvae from beach plum. *Abagrotis benjamini* adults first appeared in late June, apparently aestivating and appearing in highest numbers later in the season, primarily in August but extending through September.

**Figures 7.**
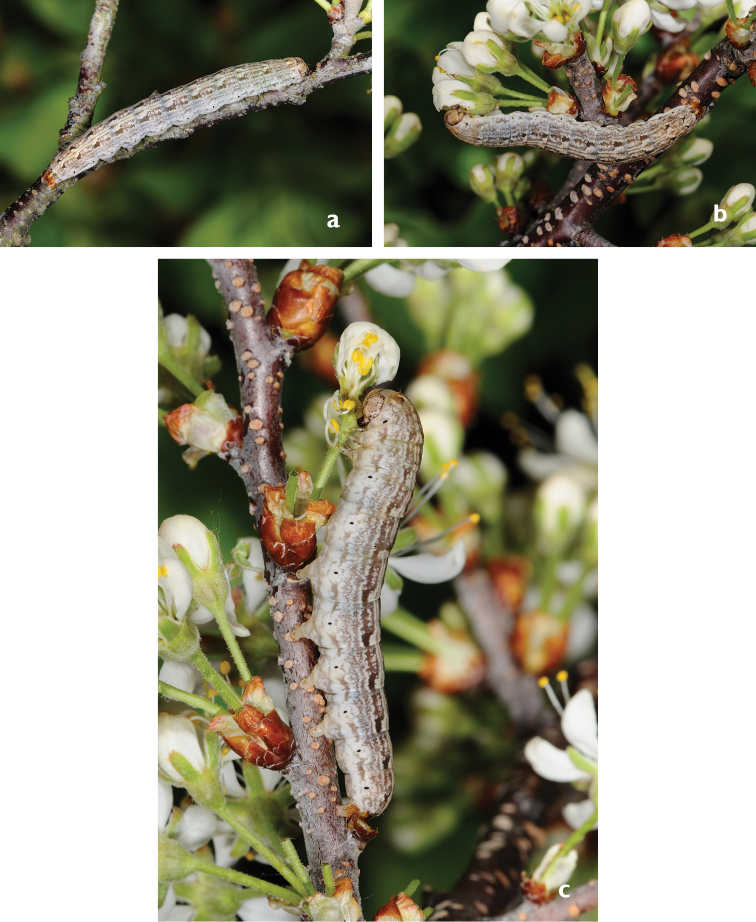
Larvae of *Sympistis riparia*. Dartmouth, MA, 2013 (M. Nelson).

**Figures 8.**
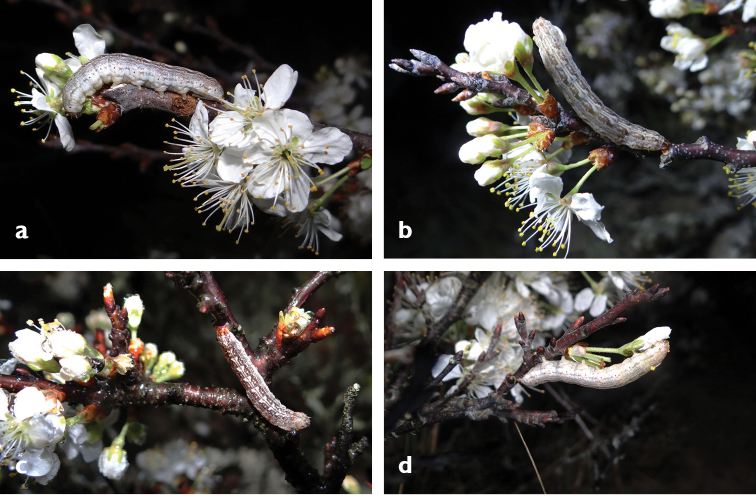
**a–d** Larvae of *Sympistis riparia* photographed *in situ*, Aquinnah, MA, 2016 (P. Goldstein).

**Figures 9.**
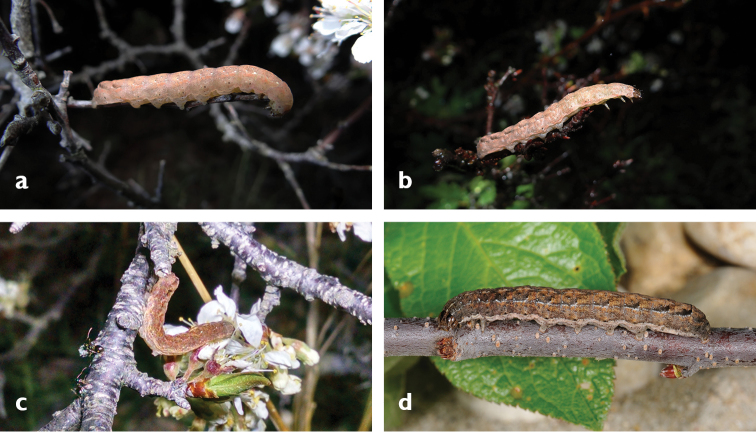
**a–d** Larvae of *Abagrotis benjamini* **a–c** *in situ*, Aquinnah, MA, 2016 (P. Goldstein) **d** Dartmouth, MA, 2012. (M. Nelson).

**Figure 10.**
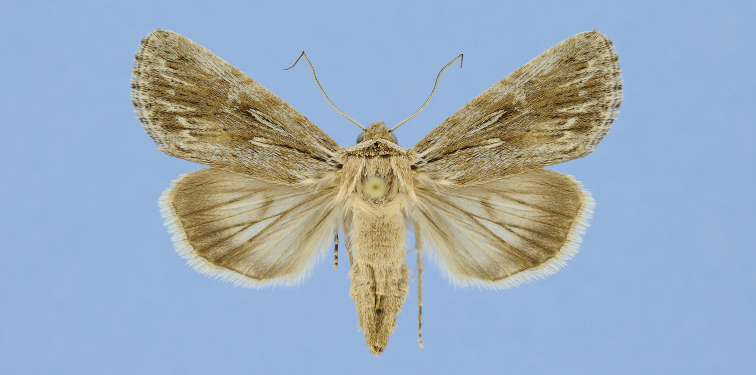
*Sympistis riparia* Morrison, Dartmouth, MA, *ex larva*. M.W. Nelson.

**Figure 11.**
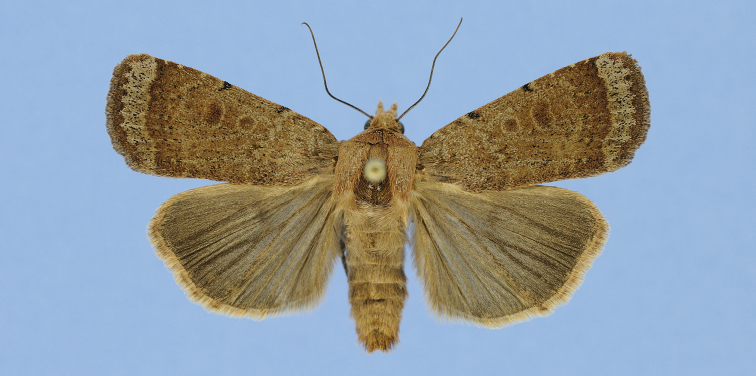
*Abagrotis benjamini* Franclemont, Dartmouth, MA, *ex larva.* M.W. Nelson.

Initial efforts to locate larvae on beach plum at Aquinnah in 2012 and 2013 were less immediately successful due to a combination of seasonal conditions and timing. Larvae of *Sympistis riparia* were collected from beach plum and reared to adults at a mainland site in Dartmouth, MA on 16 and 22 May 2013 (Fig. 7a–c) and at Aquinnah on 25 May 2015 and 18–26 May 2016 (Fig. 8a–d). At both sites, *Sympistis riparia* larvae were found alongside those of what turned out to be *Abagrotis benjamini* (Fig. 9a–d). These observations suggest a potential association of both species with beach plum, consistent with known rosaceous hosts recorded from species in both genera. Larvae were not at first found in numbers, when the beach plum was in full but early flower, suggesting a very early hatching (*Sympistis riparia*) or emergence (*Abagrotis benjamini*). A more intensive survey over seven nights in May 2016, timed to coincide with the first flowering of beach plum, documented over thirty late-instar larvae of each species. Most larvae were observed feeding on flowers and always on low growth (<1m) after 2100h. In addition to rearing larvae of *Sympistis riparia* and *Abagrotis benjamini* to adults (Figs 10, 11), we documented larvae of several other noctuines actively feeding on beach plum, including *Abagrotis alternata* and *Abagrotis magnicupida*.

Larvae of both *Abagrotis benjamini* and *Sympistis riparia* are subterranean by day, and we were impressed in particular by the ease with which *Sympistis riparia* larvae move through sand. As has been observed of other *Sympistis*, larvae of *Sympistis riparia* construct an underground cell within which to pupate. With *Sympistis riparia*, the cell is approximately 3 cm below the surface of the sand, and lined with a thin layer of silk. For this reason, the precise time of pupation was difficult to pinpoint. But, based on our extraction of some larvae and, in the case of *Abagrotis*, their successful pupation in paper towels, the pupal period for *Sympistis riparia* was under 20 days; that of *Abagrotis benjamini* was somewhat longer, between 25 and 33 days.

The maturity of the *Sympistis riparia* caterpillars at the time of their observation in the field was surprising given the brief time their host had been in flower (no more than 10 days). Some *Sympistis* (e.g., *Sympistis forbesi* Zacharczenko & Wagner, 2014, *Sympistis piffardi* (Walker, 1862)) are known to over-winter as eggs (McCabe 1985; Zacharczenko et al. 2014), others (e.g. *Sympistis perscripta* (Guenée, 1852)) as pupae (Wagner et al. 2011), but none as larvae. We suspect that larvae of *Sympistis riparia* emerge prior to the expansion and initially feed on buds, the most available meristematic tissues.

**Figure 12.**
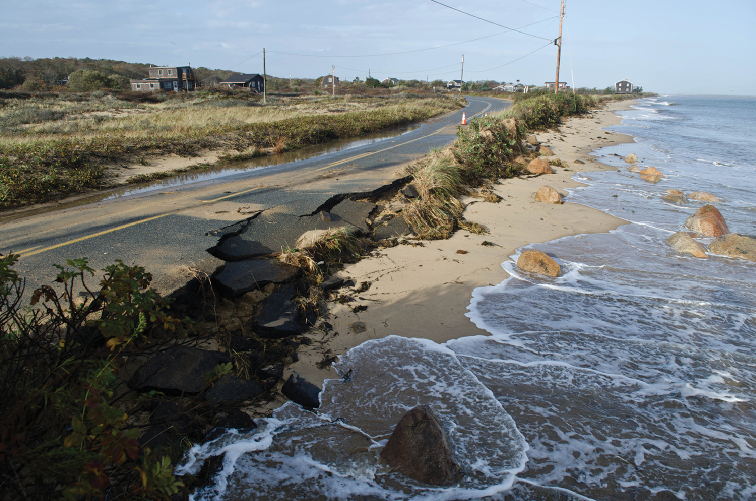
Damage from storm surge at Lobsterville Dunes following Hurricane Sandy, October 31, 2012 (A.O. Fisher).

Our observations of mature *Abagrotis* larvae, on the other hand, are consistent with those of Rings (1971, 1972a,b), Crumb (1956), and Lafontaine (1998: 206) to the effect that “most, if not all, species [of *Abagrotis*] pass the winter as partially grown larvae.” There had been some confusion concerning the diapause of *Abagrotis* in that Buckett (1968a) characterized its members as overwintering as eggs. What makes this life history especially striking in the case of Atlantic Coast dune-dwelling caterpillars, and those at this study site in particular, is the highly disturbance-prone nature of the habitat and the intensity with which it has been visited by recent storms (Fig. 12).

**Material examined.**

**Repository abbreviations.**

The following abbreviations refer to collections from which specimen material forms the basis of this work:

**AMNH** American Museum of Natural History, New York, USA

**CUIC** Cornell University Insect Collection, Ithaca, New York, USA

**UCDC** Bohart Museum, University of California, Davis, California, USA

**USNM** National Museum of Natural History [formerly, United States National Museum], Washington, District of Columbia, USA

Beyond the history of nomenclatural confusion surrounding *Abagrotis nefascia* and its synonyms, complicating matters further is the difficulty in locating type material, some of which is incompletely labeled or lost, including the genitalic preparation of the *benjamini* holotype. Notwithstanding the apparently mixed type series of *Abagrotis nefascia* (Lafontaine 1998: 234), both the holotypes of *Abagrotis crumbi* and *Abagrotis crumbi benjamini* are unaccompanied by USNM slide numbers, and since the holotype slide of *Abagrotis crumbi* was not so labeled, it is unlikely that the *Abagrotis benjamini* slide (wherever it resides) was either. The dissection described for the holotype in Franclemont’s original description (JGF 1203) appears lost, although a slide bearing the same data with an adjacent number (JGF 1202) was located.

Meanwhile, Benjamin’s dissection (FHB 850), which represents the holotype slide of *Abagrotis crumbi* as per Franclemont’s (1955) description, was originally labeled “Paratype” at the time of its preparation in 1934, more than twenty years prior, while another of Franclemont’s paratypes (FHB 843) was labeled “Holotype” by Benjamin; the name *crumbi* having apparently originated with him, based on the type material originally reared by S.E. Crumb in 1933. Two more recent preparations made by Franclemont of male *Abagrotis crumbi*, dated 14 May 1990 (JGF 7627 and 7628), are unaccompanied by specimens either at USNM or CUIC.

However, most of Franclemont’s paratype material for *Abagrotis crumbi* (24 of 27 specimens) and *Abagrotis benjamini* (5 of 8 specimens) is intact, as are series of adults and accompanying dissections of Massachusetts *Abagrotis* “*nefascia*” collected by Jones and Kimball on Martha’s Vineyard and Nantucket, the dissections made subsequently by A.E. Brower.

**Type material.**

**Figure 13.**
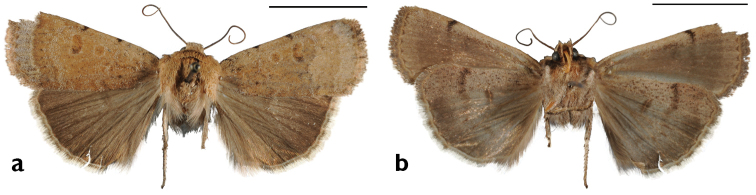
Lectotype of *Rhynchagrotis nefascia* Smith. **a** Dorsal **b** Ventral. Scale bars: 1 cm (Photos C. Richenbacher).

*Abagrotis nefascia*. (Figs 13a, b, 18). Lectotype (♂, AMNH): **New Mexico**: Ft Wingate NM VII.21; J.B. Smith Collection Rutgers; *Rhynchagrotis nefascia* ♂ type Sm.; Lectotype *Rhynchagrotis nefascia* Sm. By J.S. Buckett; ♂ Genitalia mounted on slide, F. H. R. no. 12,106; Slide labels: ♂ Genitalia *Rhynchagrotis nefascia* J. B. Smith Ft. Wingate, N.M. VII.27; No. 12,106 LECTOTYPE BALSAM Mounted XI.18.1963 Fred H. Rindge

*Abagrotis crumbi* (Figs 15a, b, 20). Holotype (♂, USNM): **Washington**: White Swan VI-5-33; White Swan No. 3; Collector S.E. Crumb; 53; 8; ♂ gen. 850 7 Feb. 34 FHB; HOLOTYPE *Abagrotis crumbi* J.G. Franclemont. Paratypes (12 of 14 ♂, 12 of 13 ♀ designated by Franclemont): **Washington**: White Swan (5♂, 6♀); Ellensburg (6♂, 3♀); Yakima (1♂, 1♀); Tieton (1♀); Cashmere (1♀)

*Abagrotis reedi* Holotype (♂, UCDC): Holotype ♂ **California**: *Abagrotis reedi* J.S. Buckett; Tecate Pk Cal San Diego Co VII-21-1963; Bill Reed Collector; 226

*Abagrotis forbesi* Holotype (♂, USNM) **Utah**: *Lampra forbesi* Benj. Holotype ♂; T Spalding IX-19-7 Stockton, Ut; Barnes Collection; Slide No. 20640.

**Other material examined.**

**Figure 14.**
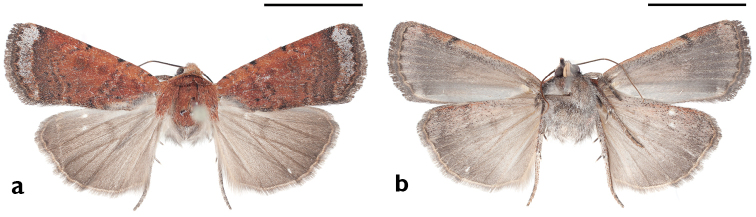
Holotype of *Abagrotis crumbi* Franclemont (=*nefascia*). **a** Dorsal **b** Ventral Scale bars: 1 cm (Photos B. Proshek).

*Abagrotis nefascia*. USA: **COLORADO** (2♀): Oak Creek Cany Col VII.12; Col. Jacob Doll.; USNM Dissection 148058♀; Glenwood Spgs Colo.; July 24–30; Barnes Collection; USNM Dissection #148049♀. **WASHINGTON** (2♂) (Figs 14a, b, 19a, b): Yakima Co. 4 mi SW Tampico 2 July 2007 BL trap, P.J. Landoldt; Barcode of Life DNA voucher specimen SampleID CCDB-28975-B03 BOLD Proc ID LNAUU1440-15; USNM Dissection 148023; USNMENT01203911; Barcode of Life DNA voucher specimen SampleID CCDB-28975-E03 BOLD Proc ID LNAUU1476-15; USNM Dissection 148024; USNMENT01203941; **WYOMING** (1♀): Converse Co. 0.3 mi S of Glenrock Mormon Canyon Rd. 7 Aug. 2002 42 51.43'N, 106 51.75'W MG Pogue at UV trap; Barcode of Life DNA voucher specimen SampleID CCDB-28975-E02 BOLD Proc ID LNAUU1475-15; USNM Dissection 148025♀; USNMENT01203940. **UTAH** (2♀): Daggett Co. Brown’s Park on Green River 1 August 2005 J.D. Hooper Coll.; UT: Daggett Co. elev. 5490' 40.54' 04.2"N, 109.08' 40.3"W; USNMENT01279055; Uintah Co. 11mi NNE of Vernal 1 Sept. 1997 @ light J.D. Hooper Coll.; Unitah Co. elev. 5850’ 40 35’ 38.8'N, 109 25’ 37.1'W; USNMENT01279080.

**Figure 15.**
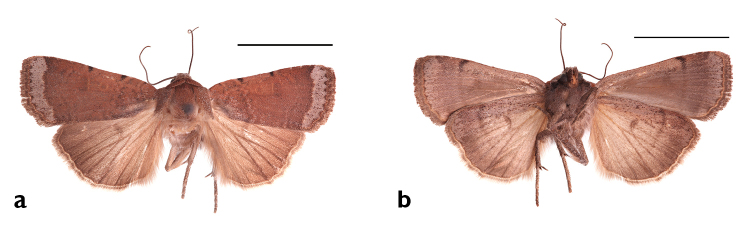
*Abagrotis nefascia* (Smith), Yakima, WA. P. Landoldt. **a** Dorsal **b** Ventral. Scale bars: 1 cm (Photos B. Proshek).

*Abagrotis magnicupida*. USA: **MASSACHUSETTS** (2♂, 2♀): West Tisbury, Manuel F. Correllus State Forest, Willow Tree Bottom, 20 July 2004; P.Z. Goldstein leg. (1♂, 3♀); USNM Dissection #148007♀; 148008♂.

*Abagrotis brunneipennis*. USA: **MASSACHUSETTS**: Edgartown, Manuel F. Correllus State Forest, bathtup deerstand 41°23.770'N, 70°35.550'W, 30 June 2011 @ UV trap P.Z. Goldstein (1♂).

*Abagrotis alternata*. USA: **MASSSACHUSETTS** (1♂, 2♀): West Tisbury, Manuel F. Correllus State Forest, Willow Tree Bottom 22 August 2000 (1♂, 1♀); USNM Dissection #148009♂; 148010♀; 20 VII 2004; USNM Dissection #148011♀.

#### Abagrotis benjamini

Taxon classificationAnimaliaLepidopteraNoctuidae

Franclemont stat. rev.

##### Material examined.

**Type material** (*Abagrotis crumbi benjamini* Franclemont, 1955)

**Figure 16.**
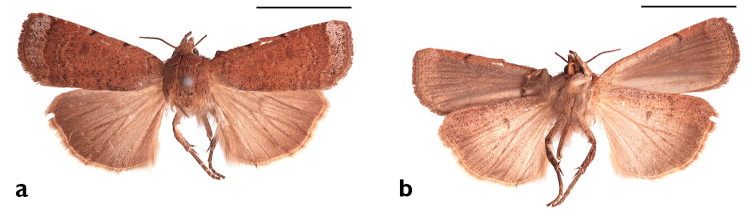
Holotype of Abagrotis crumbi race benjamini Franclemont. **a** Dorsal **b** Ventral. Scale bars: 1 cm (Photos B. Proshek).

Holotype. (Fig. 16a, b). **New York**: E.N.Y. 7.17.00; Collection BrklynMus; Acc. 17,151; ♂ Gen. #1203 F.H.B. 17 July 1935; HOLOTYPE *Abagrotis crumbi benjamini* J.G. Franclemont.

Slide labels [Paratypes]: (Fig. 22a, b) USNM 36838 ♂; ♂ Gen. #1202 *Lampra crumbi* n. sp. var. E.N.Y. A.C. Weeks 7.17.00 Coll. Brklyn Mus. F.H.B. 17 July 1935. Paratypes (2 of 5♂, 3 of 3♀ designated by Franclemont): **New York**: East New York (2♂, 1♀). **Connecticut**: East River (1♀). **Location uncertain**: “Collection J.B. Smith” (1♀).

##### Other material examined.

(not including ~13 with DNA barcodes pending)

**Figure 17.**
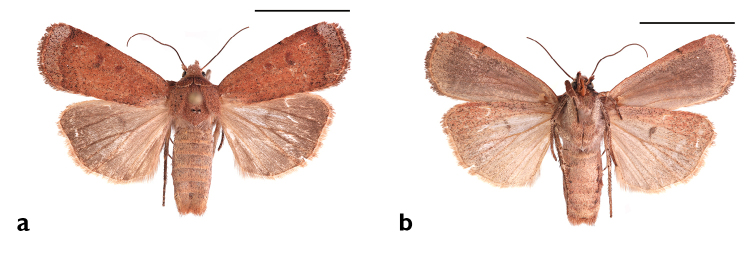
*Abagrotis benjamini* Franclemont, Aquinnah, MA. P.Z. Goldstein. **a** Dorsal **b** Ventral. Scale bars: 1 cm (Photos B. Proshek).

**MASSACHUSETTS** (28♂, 18♀): **Edgartown** (1♂, 2♀): Katama Plain 41°21.472'N, 70°31.256'W, 19 June 2010 (1♂); Wintucket Cove frost bottom 24 July 1991 (1♀); Felix Neck Wildlife Sanctuary 7 VII 1989 P.Z. Goldstein, leg. (1♀). **Aquinnah** (22♂, 15♀): East Pasture Road, 41°20.494'N, 70°47.248'W, @ UV Trap: 30 June 2013 (1♂, 1♀); 1 July 2011 (1♂); 15 July 2011 (1♂); off Moshup Trail/E. of Moshup Trail, 41.324894, -70.810698 / 41°19'29.6'N, 70°48'38.5'W (7♂, 7♀): 17 vii 2003 (3♂, 1♀) [USNM Dissection #s 148015♂; 148029♂ (Fig. 23); 148031♂]; 17 July 2015 (1♀) Barcode of Life DNA voucher specimen SampleID CCDB-28975-E01 BOLD Proc ID LNAUU 1474-15; USNM Dissection #148022♀; 31 July 2014 (1♀); 17 July 2003 (3♂, 4♀); 41°19.382'N, 70°48.66'W, 14 August 2002 (1♂); off Moshup Trail, above Zack’s Cliffs, 41°19.384'N, 70°48.655'W, (5♂, 1♀): 10 Sept 2002 (3♂); 8 sept 2011 [USNM Dissection 148028♂] (1♂); 12 Sept 2010 (1♂, 1♀); Lobsterville, 41°20'49.7'N, 70°46'54.5'W, (7♂, 6♀): 22 Sept 2012 148026♀ (1♀); 23 August 2012 (2♀); 5 July 2015 148021♂ USNMENT01203938 (1♂); 30 Aug 2014 148019♀ USNMENT01203918 (1♀) (Fig. 17a, b); 3 October 2014 148020♀USNMENT01203909 (1♀); 19 July 2012 (3♂, 1♀); 22 Sept 2012 (3♂). **West Tisbury** (5♂, 1♀): Long Point Wildlife Refuge, maritime heathland E. of Long Cove Pond 41°21.232'N, 70°37.864'W, (2♂, 1♀) 29 June 2010 @ UV trap (USNM Dissection #s 148030♂; 148056♀; 148016♂); 41°21.571'N, 70°36.031'W, 14 June 2010 @ UV trap (1♂); 41°21.572'N, 70°36.032'W, 29 August 2010 @ UV trap (1♂); Maritime heath W. of Long Cove Pond 41°21.165'N, 70°38.328'W, 10 September 2010 @ UV trap (1♂).

##### Diagnosis.

Forewing ground color ranging from pale gray brown to, more commonly, a rusty brown, occasionally reddish brown (paprika colored), never dark; both FW and soma more heavily peppered with black scales than in *Abagrotis nefascia*; both fasciae and subterminal line (as dashes on underside, scalloping on upperside) less conspicuous than in *Abagrotis nefascia*. Scobinate patch on vesicae of male genitalia with 12 or fewer denticles, at least two of them markedly larger than others. Female bursa with a single signum.

**Figure 18.**
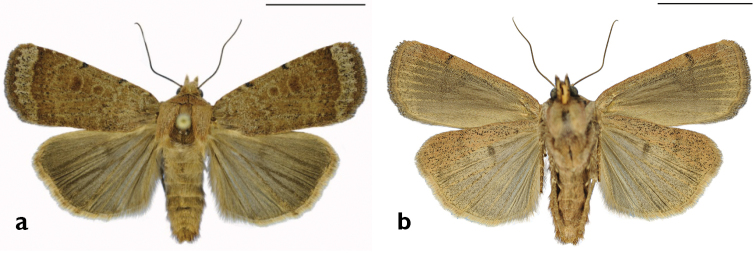
*Abagrotis benjamini* Franclemont, Dartmouth, MA. M.W. Nelson. **a** Dorsal **b** Ventral. Scale bars: 1 cm (Photos B. Proshek).

**Figure 19.**
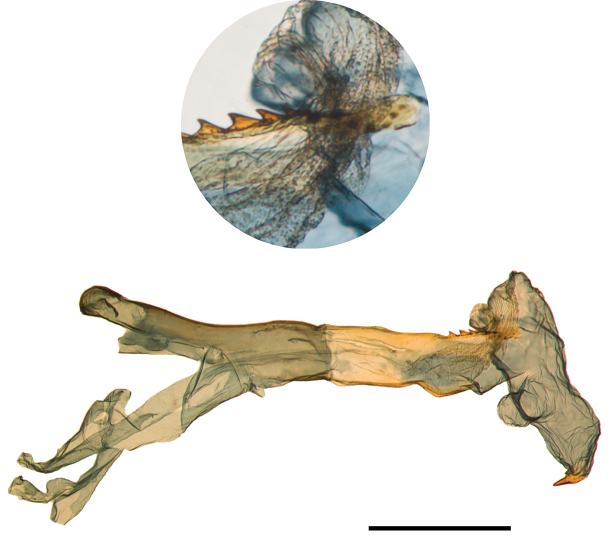
Everted vesica of *Abagrotis nefascia*, Holotype. Scale bar: 1 mm.

**Figure 20.**
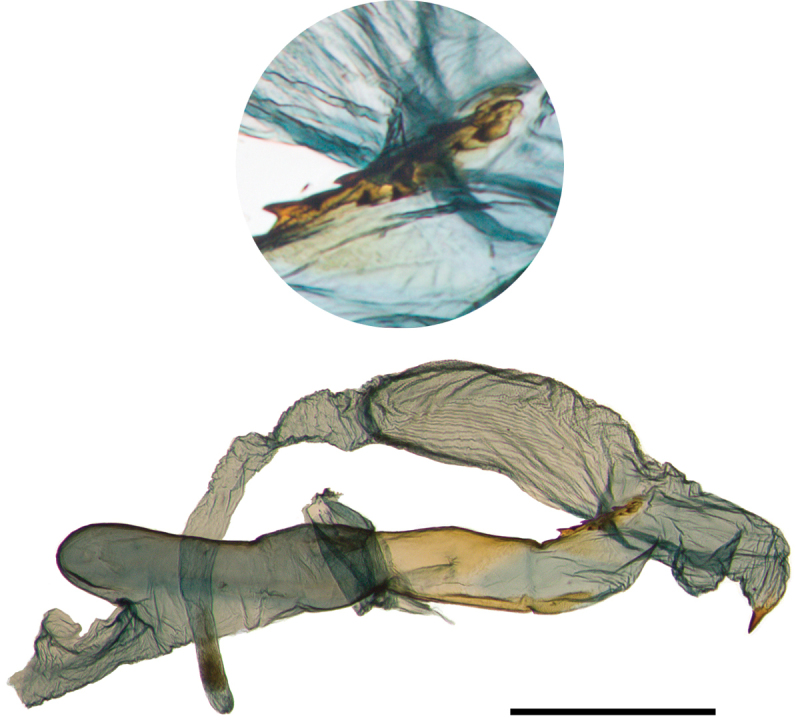
Everted vesica of *Abagrotis nefascia*, Yakima, WA. USNM dissection #s 148023, 148024. Scale bar: 1 mm.

**Figure 21.**
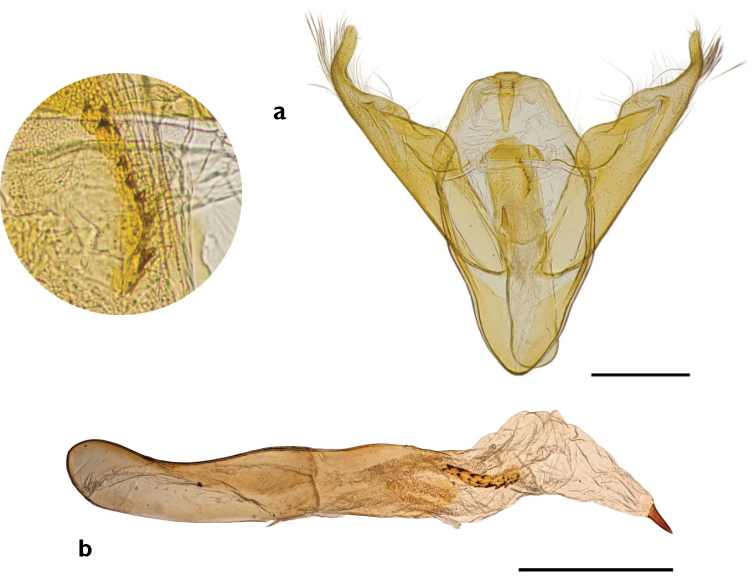
Genitalia of *Abagrotis crumbi* (=*nefascia*). **a** Holotype, ♂. USNM dissection #36819 **b** Paratype, ♂. USNM dissection #36821. Scale bars: 1 mm.

**Figure 22.**
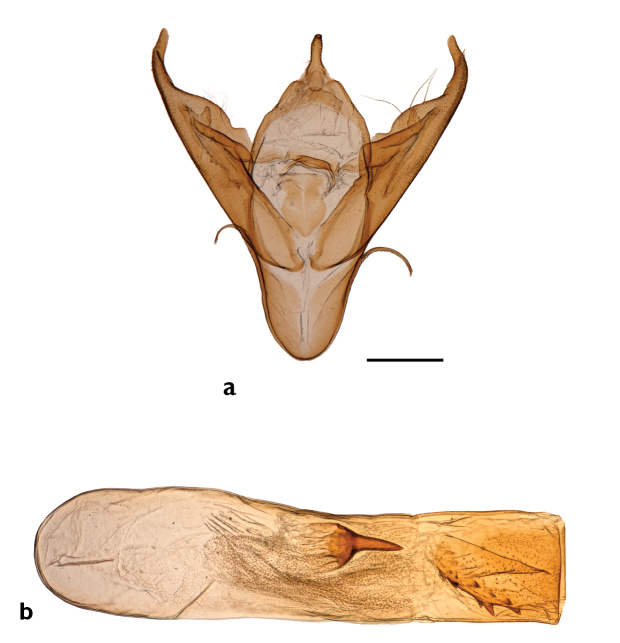
Genitalia of *Abagrotis crumbi benjamini* (Paratype). USNM dissection #36838. **a** Clasper **b** Phallus.

**Figure 23.**
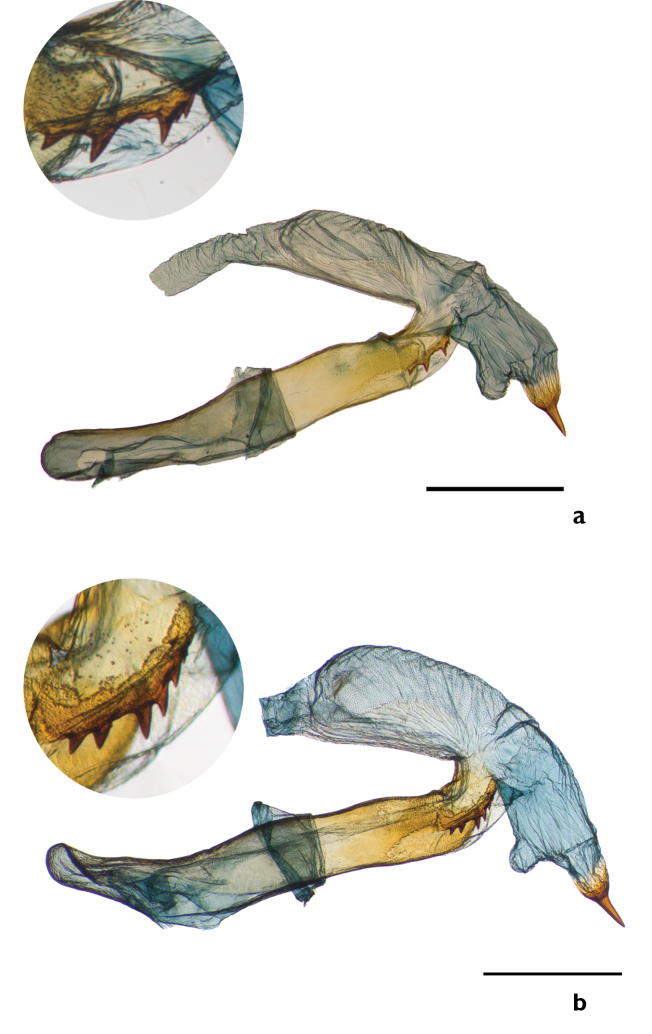
Everted vesica of *Abagrotis benjamini*, Aquinnah, MA. **a** USNM dissection #148029 **b** Everted vesica of *Abagrotis benjamini*, Dartmouth, MA. USNM dissection #148067. Cf. Figs 9d (larva), 11(adult), and 18a, b (adult).

**Figure 24.**
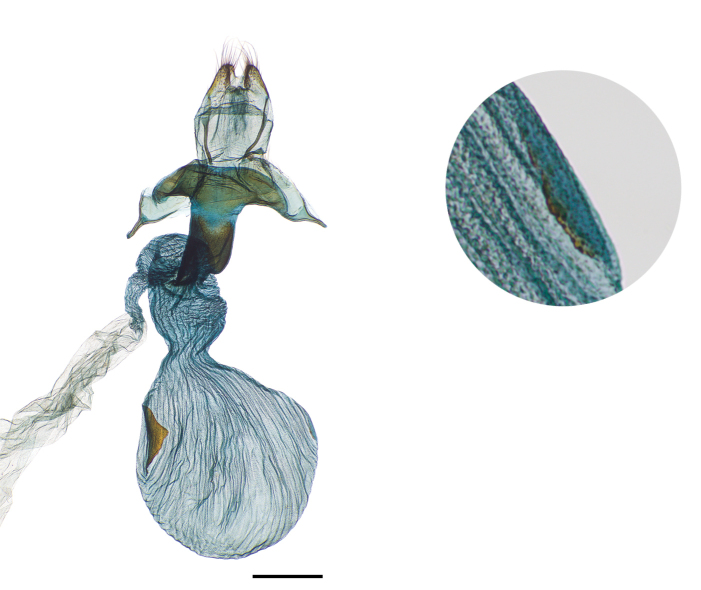
Female genitalia, *Abagrotis nefascia* USNM dissection #148066. Note presence of two signa, one reduced.

## Discussion

This work began as a seemingly challenging attempt to sleuth out what turned out to be an obvious host plant, but led us to understand better the life histories and in one case the taxonomy of two apparently psammophilic species, and to explore an interesting dune fauna that complements conventional understanding of sandplain moths. *Abagrotis benjamini* is endemic to a narrow coastal area of northeastern North America. Long assumed to be an oligophagous species associated with a range of rosaceous and ericaceous plants, *Abagrotis benjamini* is here associated specifically with beach plum *Prunus maritima* for the first time based on reared wild caught larvae, observed feeding primarily on flowers. Although the distribution of beach plum in combination with known records of *Abagrotis benjamini* and documented high abundance of the moth in dunes dominated by *Prunus maritima* strongly suggests its role as an important host, co-occurring rosaceous plants, in particular *Amelanchier* cannot be ruled out as alternative hosts. The reinstatement of *Abagrotis benjamini* follows a mildly tortuous history of nomenclatural confusion disentangled by Lafontaine (1998) but still awaiting further exploration. Of equal, if not greater interest are the specimens from British Columbia determined as *Abagrotis nefascia* Given the prominence of western *Abagrotis nefascia* among the *Abagrotis* species impacting grapes (Lowery and Mostafa 2010), further work on this complex is warranted.

The association of *Sympistis riparia* with dunes was well documented and accurate; that of *Abagrotis nefascia* with coastal heathlands less so. Our elucidation of beach plum as a host was hampered by the fact that each of these moths had been recorded from sites apparently lacking this plant, and possibly by the facultative use of other hosts, especially in the case of *Abagrotis benjamini* We were also misled by the fact that our initial target sites at Moshup Trail, while adjacent to considerable dune habitat, were more conspicuously unique for other reasons. The possibility that the unique habitat in the immediate vicinity of our trapping records had little to do with the high numbers of *Sympistis riparia* did not at first occur to us, particularly since beach plum was not especially dominant; in fact, the heathlands at the site were consistent with what we thought we knew about *Abagrotis benjamini*.

In light of a growing body of literature devoted to desert biodiversity, including that of moths (e.g., Metzler 2014), dunes may represent an underappreciated habitat for insects and biodiversity in general. Perhaps because the habitat associations of moths are not typically highly constrained, coupled with the vagility of the adult stage, dunes may be overlooked as primary natural areas for Lepidoptera, just as common plants may be under-appreciated as locally important hosts for narrowly distributed organisms. The ecological overlap with and geographic proximity of dunes to other threatened natural communities enhances their services as refugia, and we have noted concentrations of species nevertheless thriving in them. Additionally, the high abundance of species with host plants that are infrequent in dune habitat implies an importance of soils and vegetative structure that may transcend floristic composition, and challenge our assessments of habitat suitability.

## Supplementary Material

XML Treatment for Abagrotis benjamini
